# Optimal Heart Rate and Prognosis in Patients with Cardiac Amyloidosis

**DOI:** 10.3390/jcdd8120182

**Published:** 2021-12-12

**Authors:** Toshihide Izumida, Teruhiko Imamura, Makiko Nakamura, Koichiro Kinugawa

**Affiliations:** Second Department of Internal Medicine, University of Toyama, 2630 Sugitani Toyama, Toyama 930-0194, Japan; m07011ti@jichi.ac.jp (T.I.); nakamuramk1979@gmail.com (M.N.); kinugawa0422@gmail.com (K.K.)

**Keywords:** ivabradine, hemodynamics, cardiac amyloidosis

## Abstract

Background: Optimal heart rate (HR) that associates with higher cardiac output and greater clinical outcomes in patients with cardiac amyloidosis remains unknown. Methods: Consecutive patients with sinus rhythm who were diagnosed with cardiac amyloidosis at our institute between February 2015 and February 2021 were retrospectively included. Ideal HR, at which E-wave and A-wave stand adjacent without any overlaps in the trans-mitral flow echocardiography, was calculated by the formula: 86.8−0.08 × deceleration time (msec). The association between optimal HR and cardiac death or heart failure readmission was investigated. Results: Ten patients (median 74 years old, 8 men) were included. On median, actual HR was 64 bpm and ideal HR was 69 bpm. An incidence rate of the primary endpoint in the sub-optimal HR group tended to be higher than optimal HR group: one of the four patients in optimal HR group had events (25%); two of the two patients in higher HR group had events (100%); two of the four patients in lower HR group had events (50%). Conclusions: The optimal HR was associated with greater clinical outcomes in patients with cardiac amyloidosis. The clinical impact of aggressive HR optimization in this cohort remains the next concern.

## 1. Introduction

With greater progress in imaging technology and its incremental awareness, the number of patients diagnosed with cardiac amyloidosis has dramatically increased [1,2]. However, therapeutic strategy for them remains unestablished [3,4,5].

Low cardiac output, which is associated with severe diastolic dysfunction by the intramyocardial deposition of amyloid, is one of the critical comorbidities. Although not yet obviously demonstrated, a relatively higher heart rate (HR) is preferred to maintain cardiac output in such a cohort with diastolic dysfunction [3]. On the contrary, extremely high HR would rather reduce cardiac output due to incomplete relaxation. High HR further increases myocardial oxygen demand and wastes potential energy consumption [6]. Thus, optimal HR in cardiac amyloidosis remains uncertain. Optimal HR would be an important prognostic contributor, given the previous studies including those with systolic dysfunction.

Our team recently found that a “zero” overlap between E wave and A wave in the trans-mitral echocardiography flow was associated with maximum cardiac output and greater clinical outcomes in patients with systolic heart failure [7]. We expanded this concept and proposed formulas to estimate optimal HR using deceleration time alone for those with preserved ejection fraction (HFpEF) [7,8,9].

In this study, we investigated the association between optimal HR, which was calculated using a formula that was derived based on the previously proposed concept, and clinical outcomes in patients with cardiac amyloidosis.

## 2. Materials and Methods

### 2.1. Patient Selection

Consecutive patients with sinus rhythm who were diagnosed with cardiac amyloidosis at our institute between February 2015 and February 2021 were retrospectively included in this study. During the index hospitalization, patients were diagnosed with cardiac amyloidosis according to the guidelines-recommended criteria including endo-myocardial biopsy, 99mTc-pyrophosphate scintigraphy, and hematology examination [10]. All patients were diagnosed with endo-myocardial biopsy-proven cardiac amyloidosis. Patients with data deficiency, lost follow-up, dependent on hemodialysis, on pacemaker rhythm, or under mechanical circulatory supports were excluded. All patients provided written informed consent. The study protocol was approved by the institutional review board.

### 2.2. Study Protocol

Patients received transthoracic echocardiography at the time of index discharge (defined as day 0). Ideal HR was calculated using a formula as detailed below. A difference between actual and ideal HRs was calculated. Patients were classified into three groups based on the HR difference: (1) optimal HR group (HR difference ≤ 10 bpm), (2) lower HR group (HR difference < −10 bpm), and (3) higher HR group (HR difference > 10 bpm). Patients were followed from the index discharge until August 2021. The primary concern was the association between HR difference and cardiac death (cardiovascular death or cardiac sudden death) or heart failure readmission.

### 2.3. Echocardiographic Assessment

**Figure 1:**
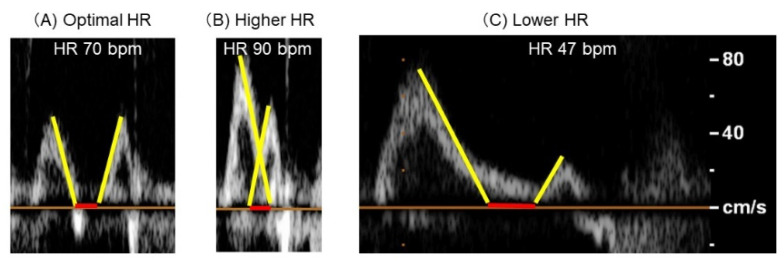
Examples of the overlap between E-wave and A-wave observed in Doppler trans-mitral echocardiographic flow. In a patient with optimal HR (HR difference ≤ ±10 bpm), E-wave and A-wave almost stand adjacent without any overlap (**A**). In a patient with higher HR (HR difference > 10 bpm), 2 waves stand overlapped (**B**). In a patient with lower HR (HR difference < −10 bpm), 2 weaves stand apart (**C**). HR; heart rate.

The expert sonographers performed transthoracic echocardiography and measured standard echocardiography parameters following the current American Society of Echocardiography guidelines [11]. Left ventricular ejection fraction (LVEF) was calculated using the modified Simpson’s method. The deceleration time of E-wave was measured in a standard manner. The overlap time between E-wave and A-wave was measured. The overlap time was expressed as a negative value in the case when two waves stand apart. Examples of different overlap patterns are displayed in Figure 1A–C.

### 2.4. Data Collection

Baseline data including demographics, comorbidities, laboratories, and echocardiographic data at index discharge were collected from the retrospective chart review. Cardiac death and heart failure readmissions that required IV-diuretics during the in-hospital careful observation were counted as primary outcomes.

### 2.5. Statistical Analysis

Continuous variables were expressed as median and interquartile and categorical variables were expressed as numbers and percentages. A formula to calculate ideal HR was constructed as followed. Multiple linear regression analysis was performed for actual HR and deceleration time to estimate the measured overlap. Considering beta-values of each variable, the equation to estimate the overlap using actual HR and deceleration time was created. By substituting zero into the equation, we constructed a formula to estimate ideal HR by using deceleration time alone. Statistical analyses were performed using SPSS Statistics 22 (IBM, Chicago, IL, USA).

## 3. Results

### 3.1. Baseline Characteristics

A total of 10 patients (74 years old, 8 men) were included (Table 1). Eight patients were diagnosed with transthyretin amyloidosis and the other two patients were light-chain amyloidosis. Four patients had New York Heart Association functional class III/IV. Plasma B-type natriuretic peptide was 350 (109,446) pg/mL. Left ventricular end-diastolic diameter was 44 (41, 46) mm and LVEF was 63% (55%, 69%). Mean right atrial pressure was 6 (4, 7) mmHg, pulmonary capillary wedge pressure was 16 (10, 21) mmHg, and cardiac index was 2.6 (2.3, 2.9) L/min/m^2^.

### 3.2. Constructing a Formula for Estimating Ideal HR

**Figure 2:**
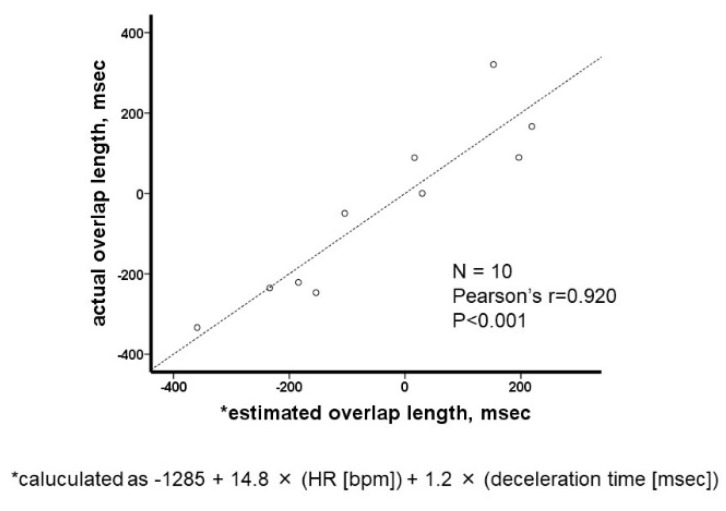
Agreement of the estimated overlap length and the actually measured overlap length. HR; heart rate.

On the basis of the multivariable linear regression analysis (both deceleration time and actual HR had *p* < 0.05), an overlap length was estimated as follows: −1285 + 1.2 × (deceleration time [msec]) + 14.8 × (actual HR [bpm]). The estimated overlap length had a significant correlation with the actual overlap length (r = 0.920; Figure 2). Based on the previous results that optimal cardiac output was obtained when E-wave and A-wave stand just adjacent, we substituted “zero” into the estimated overlap length. A formula to calculate ideal HR was constructed as follows: 86.8 − 0.08 × (deceleration time [msec]). We used this formula to calculate the ideal HR in this study.

### 3.3. HR Assessment at Index Discharge

**Figure 3:**
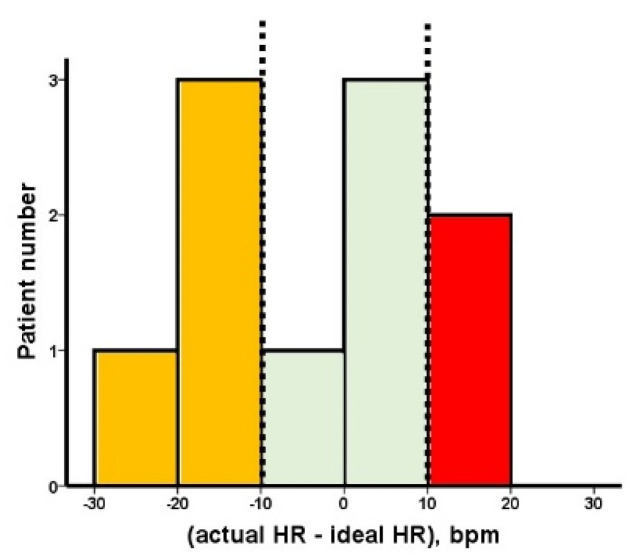
Distribution of the difference between actual HR and ideal HR. Yellow bars indicate the lower HR group: (actual HR − ideal HR) < −10 bpm. Blue bars indicate optimal HR group: −10 bpm ≤ (actual HR − ideal HR) ≤ 10 bpm. Red bars indicate the higher HR group: (actual HR − ideal HR) > 10 bpm. HR; heart rate.

Actual HR was 64 (53, 76) bpm and theoretically calculated ideal HR using the above formula was 69 (62, 75) bpm. The distribution of HR difference between actual and ideal HRs is displayed in Figure 3. Four patients had optimal HR, two patients had higher HR, and four patients had lower HR.

### 3.4. Comparison in Baseline Characteristics

As shown in Table 1, plasma B-type natriuretic peptide, cardiac troponin I, and left ventricular mass index in optimal HR group was lower than in other groups. The cardiac output measured by right heart catheterization in optimal HR group was higher than in lower HR group and lower than in higher HR group.

### 3.5. Clinical Outcomes

**Figure 4:**
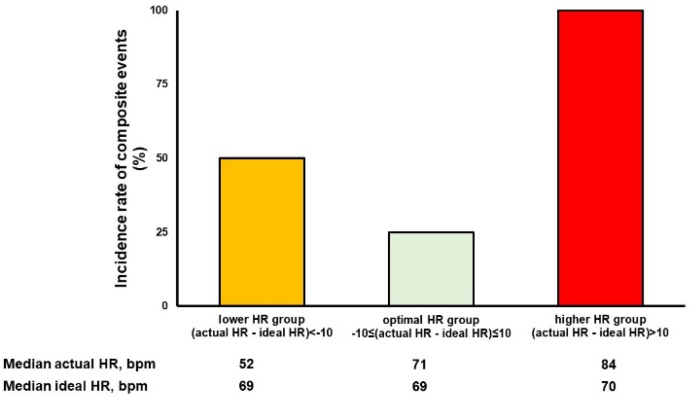
The incidence rate of primary endpoints stratified by the HR difference. The lower HR group: (actual HR − ideal HR) < −10 bpm; the optimal HR group: −10 bpm ≤ (actual HR-ideal HR) ≤ 10 bpm; the higher HR group: (actual HR − ideal HR) > 10 bpm. HR, heart rate.

During a median follow-up period of 327 [130,963] days, there were one sudden death and four readmissions due to heart failure. An incidence rate of the primary endpoint in the sub-optimal HR group tended to be higher than optimal HR group: one of the four patients in optimal HR group had events (25%); two of the two patients in higher HR group had events (100%); two of the four patients in lower HR group had events (50%) (Figure 4).

### 3.6. Tafamidis and Clinical Events

Of eight patients with transthyretin amyloidosis, 6 patients (75%) took tafamidis. The distribution of tafamidis use among the three groups was not likely to be different (three patients (75%) were in optimal HR group, two patients (50%) in lower HR group, and one patient (50%) in higher HR group). Three of the six patients with tafamidis had clinical events. There was one patient in each group.

### 3.7. Changes of Plasma B-Type Natriuretic Peptide and Cardiac Output

Plasma B-type natriuretic peptide decreased only in optimal HR group at follow-up. On the other hand, plasma B-type natriuretic peptide increased significantly in higher HR group. As for cardiac output, there was no change in follow-up in optimal HR group, an increase in lower HR group, and a decrease in higher HR group (Supplementary Figures S1 and S2).

### 3.8. A case of Aggressive HR Modulation

We had a 74-year-old woman who was diagnosed with transthyretin amyloidosis. She received pacemaker implantation to treat paroxysmal complete atrio-ventricular block. Pacing rate was 80 bpm. The overlap between the two waves was 120 msec, cardiac output was 1.52 L/min, and plasma B-type natriuretic peptide was 430 pg/mL.

One year later, the pacing rate was decreased to 70 bpm. Furthermore, two months later, the overlap between the two waves was −20 msec, cardiac output was 2.03 L/min, and plasma B-type natriuretic peptide was 273 pg/mL. In summary, following the HR optimization, cardiac output increased and plasma B-type natriuretic peptide decreased.

## 4. Discussion

In this proof-of-concept study, we investigated the association between optimal HR and the clinical outcomes following the index discharge in patients with cardiac amyloidosis. As a whole, actual HR was lower than the ideal HR, at which both E-wave and A-wave stand adjacent without any overlap (64 bpm versus 69 bpm). HR difference between actual HR and ideal HR distributed widely. Some patients had lower HR than the ideal HR, whereas others had higher HR than the ideal HR. The optimal HR, which was defined as HF difference within 10 bpm, was associated with lower mortality or heart failure recurrence following the index discharge. In addition to the higher HR, the lower HR was associated with poorer clinical outcomes.

### 4.1. Cardiac Amyloidosis and HR

Given that patients with cardiac amyloidosis showed small changes in stroke volume relative to changes in peak oxygen consumption at reaching predicted-maximal HR during the cardiopulmonary exercise test, extremely high HR could be harmful. A cohort suggested that higher HR was a predictor of worse survival in patients with cardiac amyloidosis [12]. On the contrary, considering low cardiac output due to severe diastolic dysfunction in patients with cardiac amyloidosis, extremely low HR had better be avoided to maintain cardiac output [3]. In fact, our baseline data showed that in lower HR group, cardiac output was lower and pulmonary capillary wedge pressure was higher than in other groups.

### 4.2. HR and Clinical Outcomes

Our results suggested that, only in optimal HR group, cardiac output was preserved and cardiac unloading occurred in the long-term follow-up. About half of all patients had advanced heart failure with New York Heart Association functional class III/IV. These patients might be refractory to tafamidis [13].

We propose HR modulation as another therapeutic target, particularly for such a severe cohort. We believe that the ideal HR should vary in each disease or even in each patient. This is a rationale why we propose to calculate ideal HR.

The underlying mechanisms of the association between HR and clinical outcomes in the amyloidosis cohort might be similar to those with constrictive pericarditis, given that both diseases have diastolic dysfunction. An extremely high HR might be more likely to adversely affect pulmonary congestion and low cardiac output due to a significantly poorer relaxation capacity. On the contrary, at relatively slower HR, cardiac output would be low due to inappropriately longer diastole phase [6,14].

Overall, the ideal HR calculated in this study was 69 bpm, whereas actual HR was 64 bpm. The value of ideal HR might be relatively higher than those of patients with HFrEF (around 60 bpm in general), probably due to relatively shorter deceleration time. Therefore, aggressive HR reduction using ivabradine might not be highly encouraged in most patients with cardiac amyloidosis. In patients with extremely lower HR than the ideal HR, pacemaker implantation might be recommended to maintain cardiac output and improve clinical outcomes [15,16].

### 4.3. Limitations and Future Concerns

First, the sample size was small. This is a proof-of-concept and our findings should be validated in the larger-scale studies, although cardiac amyloidosis is a rare disease. We cannot conclude any similarities for the comparison analyses with non-significance, given the small sample size. We cannot use statistics in some of analyses due to small sample size. Second, potential confounders had better be adjusted, but we failed due to a small sample size. Third, we could not perform sub-analysis separately for light-chain amyloidosis and transthyretin amyloidosis. Forth, some laboratory data were deficient including cardiac troponin. Fifth, the formula to calculate ideal HR is based on the concept that maximal cardiac output is achieved at the ideal HR. The clinical application of our theory using ivabradine or pacing requires the accumulation of more scientific evidence. Sixth, we investigate in this study just the association and causality remains unknown.

## 5. Conclusions

The optimal HR group among patients with cardiac amyloidosis was associated with greater outcomes than the sub-optimal HR group. Clinical implication of aggressive HR optimization for those with cardiac amyloidosis remains the next concern.

## Figures and Tables

**Table 1:** Baseline characteristics.

	All (n = 10)	Lower Heart Rate (n = 4)	Optimal Heart Rate (n = 4)	Higher Heart Rate (n = 2)
**Demographics**				
Transthyretin amyloidosis	8 (80)	4 (100)	3 (75)	1 (50)
Age, years	74 (64, 84)	78 (65, 85)	76 (66, 84)	67 (59, -)
Man	8 (80)	3 (75)	3 (75)	2 (100)
Body mass index, kg/m^2^	21.2 (20.8, 22.7)	21.1 (20.2, 22.6)	21.6 (21.2, 22.4)	21.8 (19.8, -)
New York Heart Association III/IV	4 (40)	1 (25)	2 (50)	1 (50)
**Laboratory data**				
White blood cell, μL	5210 (4760, 5640)	5330 (4970, 6760)	5190 (4560, 5930)	5460 (3250, -)
Hemoglobin, g/dL	13.6 (12.8, 13.9)	13.6 (13.0, 15.3)	12.8 (11.0, 13.6)	13.9 (13.7, -)
Albumin, mg/dL	4.0 (3.4, 4.1)	3.9 (3.5, 4.5)	4.0 (3.6, 4.1)	3.7 (3.4, -)
Uric acid, mg/dL	6.0 (4.9, 8.2)	6.6 (5.8, 8.2)	5.0 (4.7, 8.4)	6.5 (4.9, -)
Creatinine, mg/dL	1.0 (0.8, 1.2)	1.0 (0.9, 1.3)	0.8 (0.6, 1.2)	1.1 (1.0, -)
Total bilirubin, mg/dL	0.7 (0.5, 1.0)	0.5 (0.4, 0.8)	0.9 (0.5, 1.3)	0.7 (0.6, -)
Asparate aminotransferase, IU/L	28 (24, 31)	30 (27, 31)	27 (21, 31)	23 (19, -)
Alanine aminotransferase, IU/L	25 (17, 31)	28 (20, 41)	26 (19, 36)	18 (15, -)
Total cholesterol, mg/dL	167 (147, 202)	152 (143, 172)	193 (152, 214)	182 (156, -)
Cardiac troponin I, pg/mL	142 (87, 229)	125 (55, -)	96 (84, -)	219 (182, -)
Plasma B-type natriuretic peptide, pg/mL	350 (109, 446)	358 (146, 448)	270 (102, 436)	324 (182, -)
**Echocardiographic data**				
Left ventricular ejection fraction, %	63 (55, 69)	60 (45, 67)	63 (57, 71)	64 (53, -)
Left ventricular end-diastolic diameter, mm	44 (41, 46)	45 (43,47)	44 (38, 46)	41 (36, -)
Left ventricular mass index, g/m2	158 (125, 179)	188 (165, 269)	125 (123, 142)	158 (156, -)
Relative wall thickness	0.46 (0.42, 0.76)	0.60 (0.44, 0.76)	0.42 (0.29, 0.47)	0.65 (0.50,-)
E/e’ ratio	16.6 (11.5, 21.1)	14.7 (10.6, -)	17.3 (12.4, 33.3)	15.4 (11.9, -)
**Right heart catheterization**				
Right artery pressure, mmHg	6 (4, 7)	6 (2, 9)	6 (3, 11)	5 (4, -)
Pulmonary artery pressure, mmHg	24 (14, 29)	26 (19, 29)	22 (13, 34)	18 (14, -)
Pulmonary capillary wedge pressure, mmHg	16 (10, 21)	17 (12, 20)	15 (7, 25)	13 (10, -)
Cardiac index, L/min/m^2^	2.6 (2.3, 2.9)	2.2 (2.0, 2.6)	2.6 (2.4, 2.9)	3.0 (2.9, -)
**Past medical history**				
Hypertension	6 (60)	2 (50)	2 (50)	2 (100)
Diabetes mellitus	0 (0)	0 (0)	0 (0)	0 (0)
Hypercholesterolaemia	3 (30)	2 (50)	0 (0)	1 (50)
**Medications**				
Beta blockers	4 (40)	2 (50)	0 (0)	2 (100)
Dose, mg	0.0 (0.0, 3.8)	0.6 (0, 5.9)	0 (0,0)	6.3 (2.5, -)
RAAS inhibitors	7 (70)	2 (50)	3 (75)	2 (100)
Mineralocorticoid receptor antagonists	4 (40)	3 (75)	1 (25)	0 (0)
**Heart rate data**				
Actual heart rate, bpm	64 (53, 76)	52 (49, 57)	71 (58, 75)	84 (77, -)
Ideal heart rate, bpm	69 (62, 75)	69 (64, 72)	69 (55, 76)	70 (63, -)
Deceleration time, msec	216 (142, 301)	216 (185, 278)	225 (135, 394)	211 (123, -)
Two-wave overlap length, msec	−25 (−238, 109)	−241 (−312, −225)	44 (−37, 63)	128 (89, -)
Heart rate difference, bpm	−3 (−13, 11)	−14 (−22, −11)	1 (−5, 8)	14 (13, -)

Data represent median (quartiles Q1–Q3), or n (%). Each group was classified as follows; the lower HR group: (actual HR − ideal HR) < −10 bpm; the optimal HR group: −10 bpm ≤ (actual HR − ideal HR) ≤ 10 bpm; the higher HR group: (actual HR − ideal HR) > 10 bpm. RAAS, renin-angiotensin aldosterone system.

## Data Availability

Data are available from the corresponding author upon reasonable requests.

## Supplementary Materials

The following are available online at https://www.mdpi.com/article/10.3390/jcdd8120182/s1, Figures S1 and S2.
