# Supplementary figures and images for: Optimal Heart Rate and Prognosis in Patients with Cardiac Amyloidosis
Source: J Cardiovasc Dev Dis. 2021 Dec 12;8(12):182. doi: 10.3390/jcdd8120182 (PMC8706408; doi:10.3390/jcdd8120182)

supplementary materials

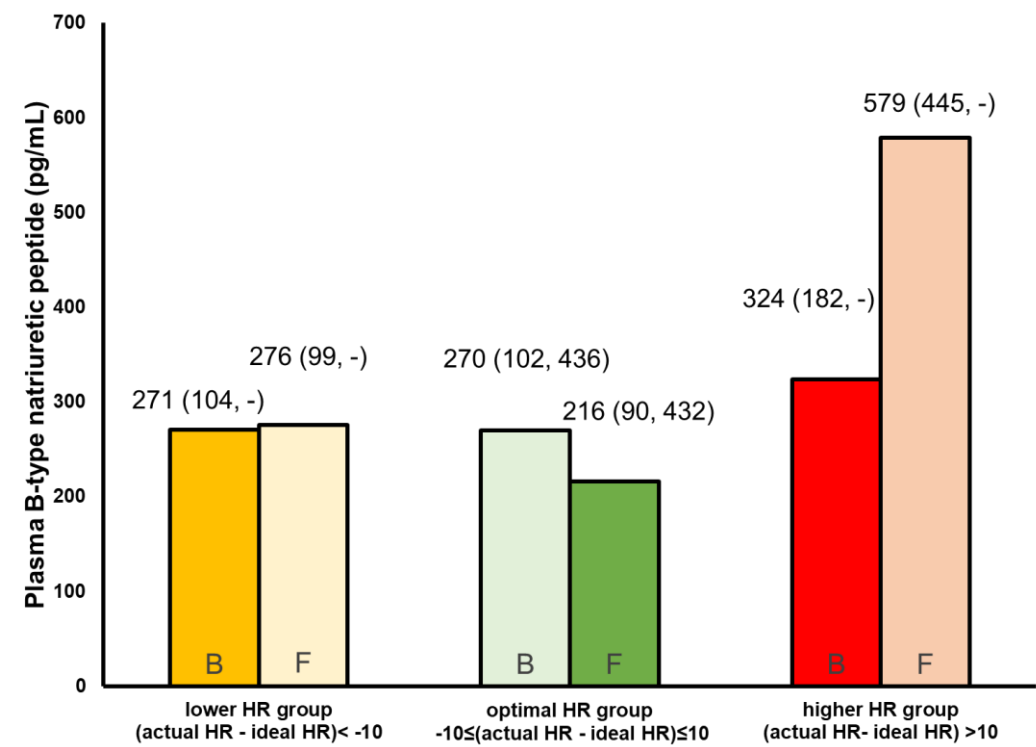

Figure S1

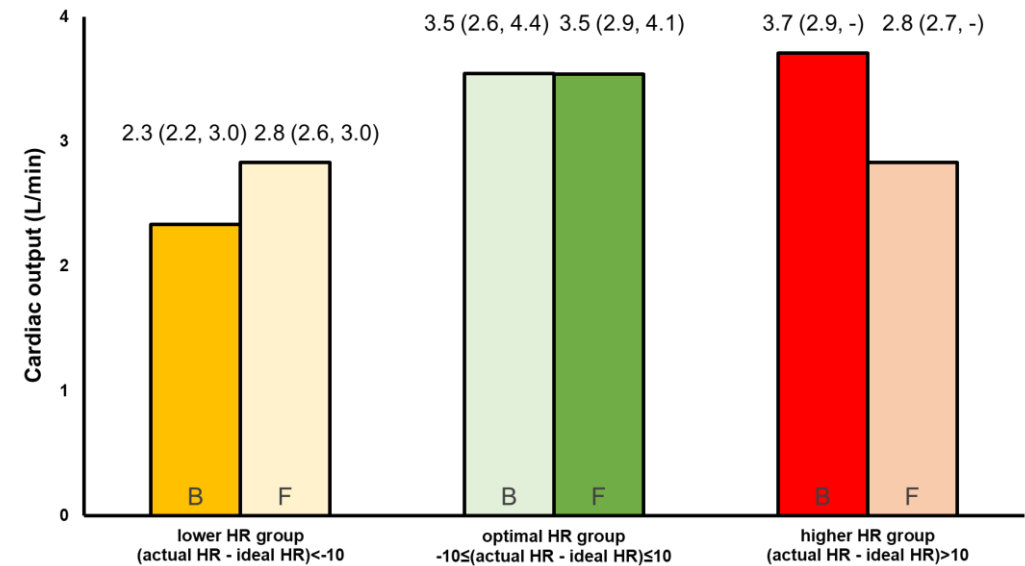

Figure S2

Supplement: Supplementary file 1 [file jcdd-08-00182-s001.zip › supplementary materials.pdf]
